# A better participant experience using sms for real time data collection

**DOI:** 10.1186/1745-6215-16-S2-O63

**Published:** 2015-11-16

**Authors:** Jamie Garner, Rhiannon Whitaker

**Affiliations:** 1 Keele University, Keele, UK

## 

High quality, error free and complete data is the Holy Grail for healthcare researchers.

Using SMS, researchers can build a data collection pathway which, when integrated with web technologies, can provide a dynamic data collection experience for each participant. It can highlight erroneous responses and missing data for correction by the participant and provide instant data delivery to the study team. The system can incorporate customisable reminders to improve overall data quality and completeness.

By using a web front end, researchers can build a dynamic workflow of questions, presenting the next item based on previous responses, skipping to the next appropriate question or to the end if appropriate. This interactive approach reduces participants’ burden and gives a more seamless data collection experience.

This data collection mechanism allows for responses to be available immediately for the study to review. The system provides a full audit trail of interactions with the participant. A dashboard enables the trial co-ordinator or data manager to visualise all the data collection points, identify non respondents and aid data cleaning in a timely fashion. Missing data or missed follow ups can then be followed up by a timely phone call and the data entered directly into the system.

SMS data collection is being used in the SCOPiC Trial (HTA 12/201/09) of stratified care in Sciatica. It is proving to be a great contribution to efficient trial conduct. Testing with a user stakeholder group generated good, positive feedback.

